# Health, well-being, and burnout amongst Early Career Doctors in Nigeria

**DOI:** 10.1371/journal.pone.0285983

**Published:** 2023-05-23

**Authors:** Akaninyene Eseme Bernard Ubom, Oladimeji Adebayo, Philip Adewale Adeoye, Kehinde K. Kanmodi, Mumeen Olaitan Salihu, Shehu Salihu Umar, Musliu Adetola Tolani, Oluwaseyi Oyekunle Ogunsuji, Henreitta I. Monye, Ugochukwu A. Eze, Yahya Abdulmajid Ibrahim, James Teri Nuhu, Temitope Toluse Selowo, Shuaibu Onoruoyiza Ibrahim, Taiwo Alatishe, Dabota Yvonne Buowari, Ukam Ekup Edadi, Adedayo Williams, Abayomi Ojo, Toba Osasona, Evo Olori Esievoadje, Taofeek Adedayo Sanni, Dare Godiya Ishaya, Abiodun Suleiman, Muhammad Sani Kabir, Ugo Uwadiako Enebeli

**Affiliations:** 1 Department of Obstetrics and Gynaecology, Obafemi Awolowo University Teaching Hospital, Ife-Ife, Nigeria; 2 Department of Medicine, University College Hospital, Ibadan, Nigeria; 3 Department of Community Medicine, Jos University Teaching Hospital, Jos, Plateau State, Nigeria; 4 Faculty of Dentistry, University of Puthisastra, Phnom Penh, Cambodia; 5 Department of Behavioural Sciences, University of Ilorin Teaching Hospital, Ilorin, Nigeria; 6 Department of Radiotherapy and Oncology, Ahmadu Bello University Teaching Hospital, Zaria, Nigeria; 7 Department of Surgery, Division of Urology, Ahmadu Bello University / Ahmadu Bello University Teaching Hospital, Zaria, Nigeria; 8 Department of Periodontology and Community Dentistry, University College Hospital, Ibadan, Nigeria; 9 Department of Ophthalmology, University College Hospital, Ibadan, Nigeria; 10 Department of Ophthalmology, Federal Medical Centre, Asaba, Nigeria; 11 Department of Ear, Nose, &Throat (ENT), Head and Neck Surgery, University of Maiduguri Teaching Hospital, Maiduguri, Borno State, Nigeria; 12 Department of Obstetrics and Gynaecology, Federal Teaching Hospital, Gombe, Nigeria; 13 Department of Chemical Pathology, Jos University Teaching Hospital, Lamingo, Jos, Nigeria; 14 Department of Anaesthesiology and Intensive Care Unit, Federal Medical Centre, Katsina, Nigeria; 15 Department of Psychiatry, LAUTECH Teaching Hospital, Ogbomosho, Nigeria; 16 Department of Accident and Emergency, University of Port Harcourt Teaching Hospital, Port Harcourt, Nigeria; 17 Department of Internal Medicine, Nephrology Unit, University of Calabar Teaching Hospital, Calabar, Nigeria; 18 Department of Anaesthesia, LTH, Osogbo, Nigeria; 19 Department of Medicine, Ekiti State University Teaching Hospital, Ido Ekiti, Nigeria; 20 Department of Medicine, Federal medical Centre, Asaba, Nigeria; 21 Department of Community Medicine, Federal Teaching Hospital, Ido Ekiti, Nigeria; 22 Department of Internal Medicine, Abubakar Tafawa Balewa University Teaching Hospital, Bauchi, Nigeria; 23 Department of Family Medicine, Abubakar Tafawa Balewa University Teaching Hospital, Bauchi, Nigeria; 24 Department of Community Medicine, University of Port Harcourt Teaching Hospital, Port Harcourt, Nigeria; George Mason University, UNITED STATES

## Abstract

**Background:**

Early Career Doctors (ECDs) in Nigeria are faced with many individual and systemic problems, which consequently adversely affect their health, well-being, patient care and safety.

**Objective:**

This study, the second phase of the Challenges of Residency Training and Early Career Doctors in Nigeria (CHARTING II) Study, sought to examine the risk factors and contributors to the health, well-being and burnout amongst Nigerian ECDs.

**Methods:**

This was a study of health, well-being and burnout amongst Nigerian ECDs. Outcome variables included burnout, depression, and anxiety, which were respectively assessed using the Copenhagen Burnout Inventory (CBI) and Oldenburg Burnout Inventory (OLBI), Patient Health Questionnaire (PHQ-9) depression scale, and Generalized Anxiety Disorder (GAD-7) scale. The quantitative data obtained was analysed using the IBM SPSS, version 24. Associations between categorical outcome and independent variables were assessed using chi square, with level of significance set at < 0.05.

**Results:**

The mean body mass index (BMI), durations of smoking and alcohol consumption of the ECDs were 25.64 ± 4.43 kg/m^2^ (overweight range), 5.33 ± 5.65 years and 8.44 ± 6.43 years respectively. Less than a third (157, 26.9%) of the ECDs exercised regularly. The most common disease conditions affecting the ECDs were musculoskeletal (65/470, 13.8%) and cardiovascular diseases (39/548, 7.1%). Almost a third (192, 30.6%) of the ECDs reported experiencing anxiety. Male and lower cadre ECDs were more likely than female and higher cadre ECDs to report anxiety, burnout and depression.

**Conclusion:**

There is an urgent need to prioritize the health and well-being of Nigerian ECDs, so as to optimize patient care and improve Nigeria’s healthcare indices.

## Introduction

Early Career Doctors (ECDs) are medical doctors in the cadre of pre-registration House Officers, Medical/Dental Officers below the rank of Principal Medical/Dental Officer (PMO/PDO), and Resident Doctors (RDs) in Nigeria [1,2]. Being a heterogenous group of medical practitioners in this low-middle income country, ECDs are faced with a lot of demographic, workplace-related and psychosocial problems [3,4]. Amongst the psychosocial problems is burnout. Burnout rates of as high as 51.9% have been reported amongst Nigerian ECDs, and this was found to be significantly associated with long work hours [5,6]. Work hours of Nigerian doctors are currently not regulated, with RDs bearing the brunt of these long unregulated work hours [7]. A recent study reported that Nigerian RDs work an average of 123 hours/week [7]. Overwork proportionally correlates with high levels of stress, depression, poor personal relationships, reduced quality of life, and in the extreme, mortality amongst medical doctors [8–10]. Cumulative fatigue from long work hours, is therefore, not only detrimental to patients’ safety, but deleterious to the overall well-being and health of physicians [11].

The physician’s health and well-being must be prioritized if patient care is to be optimized. Physicians are better able to connect with, interact, serve, and care for patients when they (the physicians) are in good health [12]. Addressing the health and well-being of physicians does not only benefit the physicians, but also patients and the entire healthcare system [12]. It is in line with this reality that the Physician’s Pledge has been revised, and the recent Charter on Physician Well-being made a renewed call for a partnership and commitment among medical professionals and healthcare organizations, to address the widespread problem of physician burnout, and promote a culture of well-being amongst physicians globally [12].

The long work hours, coupled with high burnout rates of Nigerian ECDs, is against the backdrop of a very low physician-to-patient ratio, limited slots/opportunities for training and career development, as well as poor renumeration [3,13]. A fallout of these, is the ongoing massive medical brain drain in the country, which if unchecked, has the potential of further worsening the already abysmal healthcare indices in the country [14,15]. There is therefore an urgent need to investigate and understand the full-scale of the problems faced by Nigerian ECDs, with a view to proffering relevant solutions, as well as address issues pertaining to their health and well-being. This need midwifed the Challenges of Residency Training and Early Career Doctors in Nigeria (CHARTING) Study, the largest, multi-centre, and multidisciplinary research on Nigerian ECDs, by the Research Collaborative Network (RCN), an ad hoc committee of NARD, established in 2018 [3]. The first phase of the Study (CHARTING I) examined the demographic, workplace and psychosocial issues affecting ECDs in Nigeria [3]. This second phase (CHARTING II) sought to examine other components of these issues, not explored in the first phase [4].

## Methods

### Study design

A cross sectional study of health, well-being and burnout amongst Nigerian ECDs. The study design have been previously described and published elsewhere [4].

### Inclusion criteria

All ECDs in Nigeria, currently working in a NARD centre (a hospital in Nigeria, whose doctors belong to NARD), and who consent to participating in the study.

### Exclusion criteria

All non-ECDs in Nigeria, and ECDs in Nigeria who are not working in a NARD centre.

### Study site/location

This study was conducted among 76 public tertiary hospitals in Nigeria, from which NARD derives her membership.

### Sampling method

A multi-stage sampling technique was used to recruit consenting participants in selected departments in the participating centres. The levels of sampling included:

- 31 centres were selected amongst the 76 NARD centres, across the six geopolitical zones of the country, viz: North West- 5, North Central- 7, North East- 3, South South- 5, South West- 9, and South East- 2;
- 5–10 departments were selected in each of the selected 31 centres, in such a manner that accommodated the study sample size;
- All willing and qualified participants in the selected departments of the 31 centres were recruited.

The participants were recruited from the selected departments in the participating centres. Participation was voluntary.

### Sample size

A total of 629 ECDs were included in this report. This was based on the expected frequency of 50%, to accommodate the broad and non-availability in some instances, of the prevalence rate of some of the issues to be explored. A confidence interval of 5% was used, and the design effect was set at 4, based on the 31 clusters of the survey. The sample size was calculated using StatCalc of Epi Info 7^®^, produced by the Centres for Disease Control and Prevention.

### Data collection tools/procedure

Similar to CHARTING I Study [1] the data collection tools included purpose-designed, structured questionnaires [3].

### Data analysis

#### Quantitative data

The outcome variables included burnout, depression, and anxiety. Burnout was assessed using the Copenhagen Burnout Inventory (CBI) and Oldenburg Burnout Inventory (OLBI) [16]. The CBI consists of three sub-scales measuring personal burnout (6 items), work-related burnout (7 items), and client-related burnout (6 items) [11]. Twelve of the 19 items are rated along a five-point Likert scale according to responses of frequency from ‘100 (always)’ to ‘0 (never/almost never)’. The remaining seven items rate the response according to an intensity which ranges from ‘to a very low degree’ to ‘to a very high degree’. An item in the work-related burnout subscale requires inverse scoring and the item is: *“do you have enough energy for family and friends during leisure time*?*”* The level of burnout is classified according to the total scores obtained. A score of 0–50 implied ‘no/low’ burnout, 50–74, ‘moderate’ burnout, 75–99, ‘high’ burnout, and 100, ‘severe’ burnout [11].

Oldenburg Burnout Inventory measures burnout with two dimensions: exhaustion and disengagement [12]. The dimensions are evaluated using 16 items: 8 items measure exhaustion, and 8 items measure disengagement from work. Both dimensions are evaluated by four positively worded items and four negatively worded items. Items are scored by using a scale ranging from 1 to 4 (Strongly agree–Strongly disagree). Respondents were considered to be at high risk of burnout if they met the cut-offs of 2.1 and 2.25 for the exhaustion and disengagement subscales [12].

Depression was assessed using the Patient Health Questionnaire (PHQ) depression scale, which evaluates the severity of depression using 9 items (hence called PHQ-9), which consists of the actual nine criteria on which the diagnosis of DSM-IV depressive disorders is made [13]. Each of the 9 DSM-IV criteria is scored ‘0’ (not at all) to ‘3’ (nearly every day). Question 9 is a single screening question on suicide risk. A respondent who answers ‘yes’ to question 9 needs further assessment for suicide risk by an individual who is competent to assess this risk. Total scores of 5, 10, 15, 20 represent cut-offs for mild, moderate, moderately severe, and severe depression [13].

Anxiety was assessed using a 7-item Generalized Anxiety Disorder scale (GAD-7) [14]. Each of the 7 items is scored ‘0’ (not at all), ‘1’ (several days), ‘2’ (more than half the days), and ‘3’ (nearly every day), giving a total score of 21. Scores of 5, 10 and 15 are taken as the cut-off points for mild, moderate and severe anxiety respectively [14].

This data obtained was analysed using the IBM SPSS, version 24. Categorical variables were expressed as frequency and percentage while continuous variables were expressed as mean and standard deviation or median and interquartile range (IQR) as appropriate. Associations between categorical outcome and independent variables were assessed using chi-square. Independent samples t-test and Mann Whitney U test were used to compare the variables between two categories while Kruskal Wallis test and ANOVA was used to analyse continuous variables with more than three groups. P-value < 0.05 was significant.

### Ethical considerations

This study, is part of the second phase of the Challenges of Residency Training and Early Career Doctors in Nigeria (CHARTING II) Study, and approval was obtained from the National Ethics Review Committee of the Nigerian Federal Ministry of Health with approval number: NHREC/01/01/2007-26/06/2019 [4]. Informed consents were also obtained from all the study participants, after diligently explaining the study objectives and rationale to them. The questionnaires were anonymous, and the database was passworded, accessible to only approved members of the research team.

### Funding

Authors Oladimeji Adebayo and Ugo Uwadiako Enebeli received funding for this work, from Nigerian Association of Resident Doctors (NARD)—NARD Grant 001. Oladimeji Adebayo, Dare Godiya Ishaya, Abiodun Suleiman, Muhammad Sani Kabir and Ugo Uwadiako Enebeli are former and current officials of NARD. The role of NARD did not go beyond funding.

## Results

### Sociodemographic characteristics of Nigerian ECDs

The mean age of the ECDs in our study was 32.81 ± 5.37 years. Most of them were males (413, 65.7%), married (346, 55.0%), and Registrars (280, 44.5%), with mean years of medical practice of 6.55 ± 4.22 years. These sociodemographic characteristics are shown in Table 1.

**Table 1:** Sociodemographic characteristics of ECDs in Nigeria.

Characteristics		Frequency *(n = 629)*	Percentage (%)
*Age (years)*			
Mean ± SD	32.81 ± 5.37		
Median (Range)	32 (29–36)		
*Sex*			
	Female	208	33.1
	Male	413	65.7
	No response	8	1.3
*Marital status*			
	Married	346	55.0
	Single	270	42.9
	Separated	5	0.8
	No response	8	1.3
	House officer	137	21.8
	Medical Officer	56	8.9
	Senior Medical Officer	8	1.3
	Registrar	280	44.5
	Senior Registrar	134	21.3
	No response	14	2.2
*Years of practice*			
Mean ± SD	6.55 ± 4.22		
Median (Range)	6 (4–10)		

### Lifestyles and current health status of Nigerian ECDs

The mean body mass index (BMI) of the ECDs was 25.64 ± 4.43 kg/m^2^ (overweight range). Female ECDs were significantly more likely to be overweight compared with male ECDs (26.24 ± 5.27 kg/m^2^ vs. 25.34 ± 3.96 kg/m^2^; p = 0.033). The ECDs reported mean durations of smoking and alcohol consumption of 5.33 ± 5.65 years and 8.44 ± 6.43 years respectively. Less than a third (157, 26.9%) of them exercised regularly.

There were significantly more male ECDs than female ECDs who smoked (95.7% vs. 4.3%; p = 0.006), drank alcohol (75.6% vs. 24.4%; p = 0.029), and exercised regularly (72.6% vs. 27.4%; p = 0.030). There was a significant relationship between cadre of the ECDs and their BMI (p<0.001), with Senior Medical Officers (SMOs) having higher BMI compared with Medical Officers (MOs) (28.23 ± 6.13 kg/m^2^ vs. 25.02 ± 4.25 kg/m^2^), and Senior Registrars (SRs) having higher BMI than Registrars (26.68 ± 4.07 kg/m^2^ vs. 25.84 ± 4.57 kg/m^2^). House Officers (HOs) and MOs were significantly more likely than SMOs to engage in regular exercises (19.7% vs. 10.8% vs. 1.9%; p = 0.033); so also, were Registrars significantly more likely to engage in regular exercises compared with SRs (43.9% vs. 23.6%; p = 0.033). Though not significantly, Registrars were also more likely than SRs to smoke (43.5% vs. 26.1%: p = 0.436) and consume alcohol (43.5% vs. 28.2%; p = 0.269).

The most common disease conditions affecting the ECDs were musculoskeletal (65/470, 13.8%) and cardiovascular diseases (CVDs) (39/548, 7.1%). Hypertension was the most commonly reported CVD (23/39, 59.0%), while the most common musculoskeletal condition was low back pain, reported by 65.6% of the respondents. More than one-half of the ECDs with hypertension agreed that work-related stress worsened their blood pressure control (20/39, 51.3%), whereas they achieved better blood pressure control during vacations (21/39, 53.8%). The majority of ECDs with CVDs were diagnosed within their years of medical practice (31/39, 79.5%).

Though not significantly, there were more males than females with CVDs (61.5% vs. 38.5%; p = 0.181). There were significantly more males than females with chronic respiratory diseases (65.5% vs. 43.5%; p = 0.02) and malignancies (87.5% vs. 12.5%; p = 007). There were significant relationships between cadre of the ECDs and cardiovascular and chronic respiratory diseases, with younger cadre ECDs being more affected with these diseases compared with higher cadre ECDs. Medical Officers were significantly more likely than SMOs to have CVDs (12.8% vs. 0%; p = 0.040); so also, Registrars were significantly more likely to have CVDs compared with SRs (46.2% vs. 33.3%; p = 0.040). Senior Medical Officers had significantly less chronic respiratory diseases compared with MOs (0% vs. 20.7%; p = 0.044), and SRs, less than Registrars (10.3% vs. 58.6%; p = 0.044). These findings are shown in Tables 2–4.

**Table 2:** Lifestyles and current health status of Nigerian ECDs.

Characteristics		Frequency	Percentage (%)
*Body Mass Index (BMI) (kg/m* ^*2*^ *)*			
Mean ± SD	25.64 ± 4.43		
Median (Range)	25.00 (22.34–28.07)		
*Smoking duration (years)*			
Mean ± SD	5.33 ± 5.65		
Median (Range)	4.5 (1.75–6.25)		
*Alcohol consumption duration (years)*			
Mean ± SD	8.44 ± 6.43		
Median (Range)	6 (4–12)		
*Regular exercises (n = 584)*			
	Yes	157	26.9
	No	427	73.1
*Cardiovascular disease (n = 548)*			
	Yes	39	7.1
	No	509	92.9
*Diabetes mellitus (n = 371)*			
	Yes	3	0.8
	No	368	99.2
*Chronic respiratory disease (n = 551)*			
	Yes	29	5.3
	No	522	94.7
*Musculoskeletal disease (n = 470)*			
	Yes	65	13.8
	No	405	86.2
*Injuries from accidents (n = 533)*			
	Yes	29	5.4
	No	504	94.6
*Malignancy (n = 553)*			
	Yes	8	1.4
	No	545	98.6
*Mental health illness (n = 533)*			
	Yes	19	3.6
	No	514	96.4
*Frequency of hospital admissions*			
Mean ± SD times	0.18 ± 0.47		

**Table 3:** Relationship between lifestyles and current health status and gender of Nigerian ECDs.

Characteristics	Gender		*χ* ^2^	p-value
	Male	Female		
BMI (kg/m^2^)				
Mean ± SD	25.34 ± 3.96	26.24 ± 5.27		0.033*
*Smoking (n = 598)*	n = 395	n = 203	9.805f	0.006*
Yes (n = 23)	22 (95.7%)	1 (4.3%)		
No (n = 575)	373 (64.9%)	202 (35.1%)		
*Alcohol consumption (n = 583)*	n = 389	n = 194	7.111	0.029*
Yes (n = 131)	99 (75.6%)	32 (24.4%)		
No (n = 452)	290 (64.2%)	162 (35.8%)		
*Regular exercises (n = 584)*	n = 394	n = 190	14.001	0.030*
Yes (n = 157)	114 (72.6%)	43 (27.4%)		
No (n = 427)	280 (65.6%)	147 (34.4%)		
*Cardiovascular disease (n = 548)*	n = 368	n = 180	3.420	0.181
Yes (n = 39)	24 (61.5%)	15 (38.5%)		
No (n = 509)	344 (67.6%)	165 (32.4%)		
*Diabetes mellitus (n = 371)*	n = 256	n = 115	0.008f	1.000
Yes (n = 3)	2 (66.7%)	1 (33.3%)		
No (n = 368)	254 (69.0%)	114 (31.0%)		
*Chronic respiratory disease (n = 551)*	n = 368	n = 183	7.869	0.020*
Yes (n = 29)	19 (65.5%)	10 (34.5%)		
No (n = 522)	349 (66.9%)	173 (33.1%)		
*Musculoskeletal disease (n = 470)*	n = 316	n = 154	0.680f	0.939
Yes (n = 65)	44 (67.7%)	21 (32.3%)		
No (n = 405)	272 (67.2%)	133 (32.8%)		
*Injuries from accidents (n = 533)*	n = 355	n = 178	5.509	0.064
Yes (n = 29)	19 (65.5%)	10 (34.5%)		
No (n = 504)	336 (66.7%)	168 (33.3%)		
*Malignancy (n = 553)*	n = 370	n = 183	9.446f	0.007*
Yes (n = 8)	7 (87.5%)	1 (12.5%)		
No (n = 545)	363 (66.6%)	182 (33.4%)		
*Mental health illness (n = 533)*	n = 361	n = 172	0.545	0.761
Yes (n = 19)	14 (73.7%)	5 (26.3%)		
No (n = 514)	347 (67.5%)	167 (32.5%)		

**Table 4:** Relationship between lifestyles and current health status and cadre of Nigerian ECDs.

Characteristics			Cadre			*χ* ^2^	p-value
	House Officer	Medical Officer	Senior Medical Officer	Registrar	Senior Registrar		
BMI (kg/m^2^)						5.630	<0.001*
Mean ± SD	24.15 ± 3.95	25.02 ± 4.25	28.23 ± 6.13	25.84 ± 4.57	26.68 ± 4.07		
*Smoking (n = 598)*	n = 137	n = 51	n = 8	n = 270	n = 132	7.570f	0.436
Yes (n = 23)	7 (30.4%)	0 (0)	0 (0)	10 (43.5%)	6 (26.1%)		
No (n = 575)	130 (22.6%)	51 (8.9%)	8 (1.4%)	260 (45.2%)	126 (21.9%)		
*Alcohol consumption (n = 583)*	n = 134	n = 46	n = 7	n = 265	n = 131	9.405f	0.269
Yes (n = 131)	26 (19.8%)	10 (7.6%)	1 (0.8%)	57 (43.5%)	37 (28.2%)		
No (n = 452)	108 (23.9%)	36 (8.0%)	6 (1.3%)	208 (46.0%)	94 (20.8%)		
*Regular exercises (n = 584)*	n = 129	n = 48	n = 7	n = 267	n = 133	38.204	0.033*
Yes (n = 157)	31 (19.7%)	17 (10.8%)	3 (1.9%)	69 (43.9%)	37 (23.6%)		
No (n = 427)	98 (23.0%)	31 (7.3%)	4 (0.9%)	198 (46.4%)	96 (22.5%)		
*Cardiovascular disease (n = 548)*	n = 124	n = 47	n = 7	n = 242	n = 128	15.076f	0.040*
Yes (n = 39)	3 (7.7%)	5 (12.8%)	0 (0)	18 (46.2%)	13 (33.3%)		
No (n = 509)	121 (23.8%)	42 (8.3%)	7 (1.4%)	224 (44.0%)	115 (22.6%)		
*Diabetes mellitus (n = 371)*	n = 71	n = 26	n = 6	n = 177	n = 91	6.702f	0.123
Yes (n = 3)	1 (33.3%)	1 (33.3%)	0 (0)	0 (0)	1 (33.3%)		
No (n = 368)	70 (19.0%)	25 (6.8%)	6 (1.6%)	177 (48.1%)	90 (24.5%)		
*Chronic respiratory disease (n = 551)*	n = 116	n = 44	n = 7	n = 252	n = 132	14.698	0.044*
Yes (n = 29)	3 (10.3%)	6 (20.7%	0 (0)	17 (58.6%)	3 (10.3%)		
No (n = 522)	113 (21.6%)	38 (7.3%)	7 (1.3%)	235 (45.0%)	129 (24.7%)		
*Musculoskeletal disease (n = 470)*	n = 102	n = 43	n = 6	n = 212	n = 107	14.524	0.069
Yes (n = 65)	4 (6.2%)	9 (13.8%)	1 (1.5%)	37 (56.9%)	14 (21.5%)		
No (n = 405)	98 (24.2%)	34 (8.4%)	5 (1.2%)	175 (43.2%)	93 (23.0%)		
*Injuries from accidents (n = 533)*	n = 116	n = 47	n = 5	n = 237	n = 128	13.359f	0.074
Yes (n = 29)	7 (24.1%)	4 (13.8%)	0 (0)	10 (34.5%)	8 (27.6%)		
No (n = 504)	109 (21.6%)	43 (8.5%)	5 (1.0%)	227 (45.0)	120 (23.8%)		
*Malignancy (n = 553)*	n = 119	n = 46	n = 7	n = 249	n = 132	5.342f	0.703
Yes (n = 8)	2 (25.0%)	1 (12.5%)	0 (0)	4 (50.0%)	1 (12.5%)		
No (n = 545)	117 (21.5%)	45 (8.3%)	7 (1.3%)	245 (45.0%)	131 (24.0%)		
*Mental health illness (n = 553)*	n = 121	n = 46	n = 7	n = 255	n = 124	13.318f	0.073
Yes (n = 19)	2 (10.5%)	2 (10.5%)	0 (0)	14 (73.7%)	1 (5.3%)		
No (534)	119 (22.3%)	44 (8.2%)	7 (1.3%)	241 (45.1%)	123 (23.0%)		

### Work hours, burnout, anxiety, and depression amongst Nigerian ECDs

The ECDs reported mean work hours/day and call hours/week of 10.81 ± 8.70 hours and 41.80 ± 35.05 hours respectively. Even though not significantly, male ECDs reported higher mean work hours/day and call hours/week compared with female ECDs (11.21 ± 8.49 vs. 10.10 ± 9.26; p = 0.180 and 42.83 ± 37.22 vs. 39.18 ± 29.96; p = 0.253 respectively). Lower cadre ECDs (MOs and Registrars) also reported higher mean works hours/day and call hours/week compared with higher cadre ECDs (SMOs and SRs). While MOs reported mean work hours/day of 10.04 ± 6.36 hours, SMOs reported mean daily work hours of 8.86 ± 1.95 hours; p = 0.538. Registrars reported significantly higher mean call hours/week compared with SRs (40.70 ± 37.61 vs. 39.13 ± 31.86; p = 0.027).

Almost a third (192, 30.6%) of the respondents reported experiencing anxiety, out of which 22.7% met the criteria for moderate to severe anxiety using the GAD 7 Anxiety Severity scoring. Though males reported more anxiety than females, this was not statistically significant (60.9% vs. 39.1%; p = 0.066). Compared with females, males were significantly more likely to report anger (58.2% vs. 41.8%; p = 0.030), feeling of being overwhelmed (57.4% vs. 42.6%; p = <0.001), and low self-esteem (54.1% vs. 45.9%; p = 0.045). Male ECDs were also more likely than females, to report higher personal and work burnout using the CBI (12.13 ± 4.77 vs. 11.65 ± 4.25; p = 0.204 and 14.78 ± 5.46 vs. 14.62 ± 5.13; p = 0.725 respectively), desire to quit medical practice/residency (64% vs. 34%; p = 0.566), as well as suicidal ideations (69.2% vs. 30.8%; p = 1.000), though not significantly so. More than two-fifths of the ECDs (247, 42.1%) reported very bad, bad or neither good nor bad work life balance. Males were more likely than females to report very bad, bad or neither good nor bad work life balance (63.6% vs. 36.4%; p = 0.336).

Lower cadre ECDs were more likely than higher cadre ECDs to be affected by anxiety, burnout and depression. Compared with SRs, Registrars were significantly more likely to report anxiety (43.5% vs. 18.8%; p = 0.033), anger (44.3% vs. 14.5%; p = 0.005), very bad work life balance (45.5% vs. 36.4%; p = 0.001), desire to quit residency (66.7% vs. 25.8%; p<0.000), and higher mean burnout based on the OLBI (39.93 ± 6.67 vs. 37.43 ± 5.59; p = <0.001). Though not significantly, Registrars also reported more feeling of being overwhelmed (43.3% vs. 24.7%; p = 0.197), low self-esteem (49.2% vs. 15.3%; p = 0.543), higher mean PHQ-9 Depression Severity (4.36 ± 4.71 vs. 3.76 ± 4.61; p = 0.093), and suicidal ideations (41.7% vs. 8.3%; p = 0.476), compared with SRs. Significantly more Registrars than SRs reported that these feelings negatively affected their work (50.8% vs. 24.6%; p = 0.048). House officers also reported significantly higher mean client-related burnout based on the CBI cut offs compared to MOs (16.92 ± 4.67 vs. 16.18 ± 5.31; p = 0.034). Compared to SMOs, MOs had higher mean PHQ-9 Depression Severity (5.42 ± 5.32 vs. 2.00 ± 4.44; p = 0.093). These findings are shown in Tables 5 and 6.

**Table 5:** Work hours, burnout, anxiety and depression and gender differences amongst Nigerian ECDs.

Characteristics	Gender		Mann Whitney U / t-test	p-value
	Male	Female		
*Work hours per day (n = 541)*	n = 363	n = 178		
Mean ± SD 10.81 ± 8.70	11.21 ± 8.49	10.10 ± 9.26	1.343	0.180
*Call hours per week (n = 491)*	n = 340	n = 151		
Mean ± SD 41.80 ± 35.05	42.83 ± 37.22	39.18 ± 29.96	1.144	0.253
			*χ* ^2^	
*Anxiety (n = 628)*	n = 417	n = 211	3.701	0.066
Yes (n = 192)	117 (60.9%)	75 (39.1%)		
No (n = 436)	300 (68.8%)	136 (31.2%)		
*GAD 7 Anxiety Severity (n = 620)*	n = 412	n = 208	3.2758	0.347
0–5; Mild (n = 479)	324 (67.6%)	155 (32.4%)		
6–10; Moderate (n = 91)	53 (58.2%)	38 (41.8%)		
11–15; Moderately Severe (n = 43)	30 (69.8%)	13 (30.2%)		
15–21; Severe (n = 7)	5 (71.4%)	2 (28.6%)		
*Lack of motivation (n = 628)*	n = 417	n = 211	0.056	0.865
Yes (n = 272)	182 (66.9%)	90 (33.1%)		
No (n = 356)	235 (66.0%)	121 (34.0%)		
*Weeping spells (n = 628)*	n = 417	n = 211	12.461	0.001*
Yes (n = 43)	18 (41.9%)	25 (58.1%)		
No (585)	399 (68.2%)	186 (31.8%)		
*Anger (n = 628)*	n = 417	n = 211	5.125	0.030*
Yes (n = 134)	78 (58.2%)	56 (41.8%)		
No (n = 494)	339 (68.6%)	155 (31.4%)		
*Suicidal Ideations (n = 628)*	n = 417	n = 211	0.048	1.000
Yes (n = 13)	9 (69.2%)	4 (30.8%)		
No (n = 615)	408 (66.3%)	207 (33.7%)		
*A desire to quit medical practice/residency (n = 628)*	n = 417	n = 211	0.307	0.566
Yes (n = 100)	64 (64.0%)	36 (36.0%)		
No (n = 528)	353 (66.9%)	175 (33.1%)		
*Feeling of being overwhelmed (n = 628)*	n = 417	n = 211	14.885	<0.001*
Yes (n = 249)	143 (57.4%)	106 (42.6%)		
No (n = 379)	274 (72.3%)	105 (27.7%)		
*Low self-esteem (n = 628)*	n = 417	n = 211	4.584	0.045*
Yes (n = 61)	33 (54.1%)	28 (45.9%)		
No (n = 567)	384 (67.7%)	183 (32.3%)		
*Has any of the above feelings negatively affected your work*? *(n = 546)*	n = 362	n = 184	2.309	0.129
Yes (n = 196)	138 (70.4%)	58 (29.6%)		
No (n = 350)	224 (64.0%)	126 (36.0%)		
*Work Life Balance (n = 587)*	n = 391	n = 196	4.560	0.336
Very Bad (n = 11)	9 (81.8%)	2 (18.2%)		
Bad (n = 78)	52 (66.7%)	26 (33.3%)		
Neither Bad or Good (n = 158)	96 (60.8%)	62 (39.2%)		
Good (n = 234)	163 (69.7%)	71 (30.3%)		
Very Good (n = 106)	71 (67.0%)	35 (33.0%)		
			Mann Whitney U / t-test	p-value
*Copenhagen Burnout Inventory (CBI)*				
Personal Burnout (n = 615)	n = 413	n = 202		
Mean ± SD 11.95 ± 4.59	12.13 ± 4.77	11.65 ± 4.25	1.271	0.204
Work Burnout (n = 615)	n = 413	n = 202		
Mean ± SD 14.71 ± 5.33	14.78 ± 5.46	14.62 ± 5.13	0.352	0.725
Client-related Burnout (n = 609)	n = 408	n = 201		
Mean ± SD 17.51 ± 4.91	16.99 ± 5.16	18.58 ± 4.23	-3.777	<0.001*
*PHQ-9 Depression Severity (n = 629)*	n = 417	n = 212		
Mean ± SD 4.44 ± 4.85	4.39 ± 4.89	4.46 ± 4.75	-0.173	0.862

**Table 6:** Work hours, burnout, anxiety and depression and cadre differences amongst Nigerian ECDs.

Characteristics	Current Status					Kruskal Wallis / ANOVA	p-value
	House Officer	Medical Officer	Registrar	Senior Registrar	Senior Medical Officer		
*Work hours per day (n = 541)*	n = 112	n = 37	n = 256	n = 129	n = 7		
Mean ± SD 10.81 ± 8.70	11.02 ± 7.24	10.04 ± 6.36	11.37 ± 10.68	9.90 ± 6.05	8.86 ± 1.95	0.780	0.538
*Call hours per week (n = 491)*	n = 111	n = 38	n = 227	n = 108	n = 7		
Mean ± SD 41.80 ± 35.05	51.35 ± 33.81	32.55 ± 31.19	40.70 ± 37.61	39.13 ± 31.86	45.23 ± 24.73	2.775	0.027*
						*χ* ^2^	
*Anxiety (n = 622)*	n = 139	n = 56	n = 282	n = 137	n = 8	10.513	0.033*
Yes (n = 191)	57 (29.8%)	14 (7.3%)	83 (43.5%)	36 (18.8%)	1 (0.5%)		
No (n = 431)	82 (19.0%)	42 (9.7%)	199 (46.2%)	101 (23.4%)	7 (1.6%)		
*GAD 7 Anxiety Severity (n = 614)*	n = 138	n = 53	n = 280	n = 136	n = 7	7.800	0.801
0–5; Mild (n = 473)	102 (21.6%)	39 (8.2%)	216 (45.7%)	110 (23.3%)	6 (1.3%)		
6–10; Moderate (n = 92)	26 (28.3%)	7 (7.6%)	40 (43.5%)	18 (19.6%)	1 (1.1%)		
11–15; Moderately Severe (n = 42)	9 (21.4%)	6 (14.3%)	19 (45.2%)	8 (19.0%)	0 (0)		
15–21; Severe (n = 7)	1 (14.3%)	1 (14.3%)	5 (71.4%)	0 (0)	0 (0)		
*Lack of motivation (n = 622)*	n = 139	n = 56	n = 282	n = 137	n = 8	3.607f	0.467
Yes n = 270	60 (22.2%)	24 (8.9%)	132 (48.9%)	51 (18.9%)	3 (1.1%)		
No n = 352	79 (22.4%)	32 (9.1%)	150 (42.6%)	86 (24.4%)	5 (1.4%)		
*Weeping spells (n = 622)*	n = 139	n = 56	n = 282	n = 137	n = 8	1.030f	0.897
Yes (n = 42)	12 (28.6%)	3 (7.1%)	19 (45.2%)	8 (19.0%)	0 (0)		
No (n = 580)	127 (21.9%)	53 (9.1%)	263 (45.3%)	129 (22.2%)	8 (1.4%)		
*Anger (n = 622)*	n = 139	n = 56	n = 282	n = 137	n = 8	14.878	0.005*
Yes (n = 131)	44 (33.6%)	9 (6.9%)	58 (44.3%)	19 (14.5%)	1 (0.8%)		
No (n = 491)	95 (19.3%)	47 (9.6%)	224 (45.6%)	118 (24.0%)	7 (1.4%)		
*Suicidal Ideations (n = 622)*	n = 139	n = 56	n = 282	n = 137	n = 8	3.228f	0.476
Yes (n = 12)	4 (33.3%)	2 (16.7%)	5 (41.7%)	1 (8.3%)	0 (0)		
No (n = 610)	135 (22.1%)	54 (8.9%)	277 (45.4%)	136 (22.3%)	8 (1.3%)		
*A desire to quit medical practice/ residency (n = 622)*	n = 139	n = 56	n = 282	n = 137	n = 8	50.454	<0.000*
Yes (n = 132)	7 (5.3%)	2 (1.5%)	88 (66.7%)	34 (25.8%)	1 (0.8%)		
No (n = 490)	132 (26.9%)	54 (11.0%)	194 (39.6%)	103 (21.0%)	7 (1.4%)		
*Feeling of being overwhelmed (n = 622)*	n = 139	n = 56	n = 282	n = 137	n = 8	5.992f	0.197
Yes (n = 247)	60 (24.3%)	18 (7.3%)	107 (43.3%)	61 (24.7%)	1 (0.4%)		
No (n = 375)	79 (21.1%)	38 (10.1%)	175 (46.7%)	76 (20.3%)	7 (1.9%)		
*Low self-esteem (n = 622)*	n = 139	n = 56	n = 282	n = 137	n = 8	3.089	0.543
Yes (n = 59)	16 (27.1%)	5 (8.5%)	29 (49.2%)	9 (15.3%)	0 (0)		
No (n = 563)	123 (21.8%)	51 (9.1%)	253 (44.9%)	128 (22.7%)	8 (1.4%)		
*Has any of the above feelings negatively affected your work*? *(n = 540)*	n = 117	n = 45	n = 247	n = 126	n = 5	9.282f	0.048*
Yes (n = 195)	31 (15.9%)	17 (8.7%)	99 (50.8%)	48 (24.6%)	0 (0)		
No (n = 345)	86 (24.9%)	28 (8.1%)	148 (42.9%)	78 (22.6%)	5 (1.4%)		
*Work Life Balance (n = 582)*	n = 127	n = 49	n = 269	n = 130	n = 7	38.987	0.001*
Very Bad (n = 11)	0 (0)	1 (9.1%)	5 (45.5%)	4 (36.4%)	1 (9.1%)		
Bad (n = 79)	6 (7.6%)	11 (13.9%)	35 (44.3%)	25 (31.6%)	2 (2.5%)		
Neither Bad or Good (n = 153)	25 (16.3%)	15 (9.8%)	75 (49.0%)	35 (22.9%)	3 (2.0%)		
Good (n = 234)	67 (28.6%)	16 (6.8%)	105 (44.9%)	45 (19.2%)	1 (0.4%)		
Very Good (n = 105)	29 (27.6%)	6 (5.7%)	49 (46.7%)	21 (20.0%)	0 (0)		
						Kruskal Wallis / ANOVA	
*Copenhagen Burnout Inventory (CBI)*							
Personal Burnout (n = 602)	n = 134	n = 51	n = 276	n = 134	n = 7		
Mean ± SD 11.95 ± 4.59	10.33 ± 4.53	12.35 ± 4.49	11.96 ± 4.63	13.26 ± 4.21	14.86 ± 1.86	8.085	<0.001*
Work Burnout (n = 602)	n = 134	n = 51	n = 276	n = 134	n = 7		
Mean ± SD 14.71 ± 5.33	12.59 ± 5.41	15.02 ± 5.18	14.74 ± 5.25	16.67 ± 4.86	16.86 ± 2.12	10.781	<0.001*
Client-related Burnout (n = 596)	n = 133	n = 51	n = 272	n = 133	n = 7		
Mean ± SD 17.51 ± 4.91	16.92 ± 4.67	16.18 ± 5.31	17.72 ± 4.79	18.34 ± 5.13	18.57 ± 2.57	2.614	0.034*
*Oldenburg Burnout Inventory (OLBI) (n = 602)*	n = 135	n = 50	n = 276	n = 134	n = 7		
Mean ± SD 39.88 ± 6.54	42.50 ± 6.21	39.70 ± 6.66	39.93 ± 6.67	37.43 ± 5.59	34.71 ± 4.50	12.064	<0.001*
*PHQ = 9 Depression Severity (n = 615)*	n = 138	n = 53	n = 281	n = 136	n = 7		
Mean ± SD 4.44 ± 4.85	4.91 ± 5.02	5.42 ± 5.32	4.36 ± 4.71	3.76 ± 4.61	2.00 ± 4.44	2.001	0.093

## Discussion

This a large study, a few of such among ECDs in Sub Saharan Africa to evaluate their overall health. Dietary habits such as eating lunch out of home, and snacking between meals, chronic stress with little sleep, coupled with sedentary and urban lifestyles, make doctors prone to overweight and obesity [17,18]. Female gender and physical inactivity were risk factors for the prevalent overweight status of the ECDs in our study. Similar to the findings in other studies, overweight amongst female doctors may not be unconnected with their relatively more sedentary lifestyle, hormonal and physiological factors such as pregnancy, and lactation [17–19]. Our study also found that higher cadre ECDs were significantly more likely to be overweight compared to lower cadre ECDs. Aside from the fact that lower cadre ECDs were more likely than their more senior colleagues to engage in regular physical exercises, as found in this study, this finding may not be unconnected with the older age, higher incomes, and higher likelihood of being married of higher cadre ECDs, as increasing age, marital status, and higher socioeconomic status have been documented to be associated with an increased likelihood of being overweight and obesity [19,20]. More so, compared with higher cadre ECDs, lower cadre ECDs work longer hours (as seen in this study), and are therefore more physically active, thus reducing the risk of being overweight. The high prevalence of overweight amongst ECDs in our study has negative implications on their clinical practices and personal health. Overweight doctors are less likely than their normal weight counterparts to counsel/advise overweight or obese patients on weight loss strategies/healthy weight management practices [18,21]. Obesity also increases the risks of CVDs, diabetes mellitus (DM), stroke, osteoarthritis, sleep apnoea, gynaecological problems including amenorrhoea, menorrhagia, and infertility, as well as various malignancies [17,20]. This possibly explains the frequency of cardiovascular and musculoskeletal diseases, which were the most common disease conditions affecting ECDs in our study.

The higher incidence of CVDs, respiratory diseases, and cancers amongst male and lower cadre ECDs compared to females and higher cadre ECDs in our study, may not be unconnected with the higher alcohol consumption and tobacco smoking of the former compared to the latter. Smokers have a higher risk of mortality from hypertensive heart disease, respiratory diseases, and cancers, amongst others [22]. The cardiovascular health benefits of reduction in alcohol consumption, even for light to moderate drinkers, is also documented in the literature [23]. In addition, it has been demonstrated that in younger age groups, females have a lower risk of CVDs [24]. This may also possibly explain the higher incidence of CVDs amongst the male ECDs in our study.

The male ECDs in our study reported longer routine work and on-calls hours. Given that 65% of the respondents in our study were RDs, a preponderance of males in the surgical specialties, which are characterised by longer work hours, with little sleep, compared to the non-surgical specialties [6], may explain the longer routine work and on-call hours reported by the male ECDs. The higher physical demand, including long theatre hours of surgical specialties make these specialties less attractive to females [25]. There is a significant association between burnout and long work hours [4,5]. This possibly explains why male and lower cadre ECDs, who worked longer hours compared to female and higher cadre ECDs, reported higher rates of burnout. Feelings of being overwhelmed, low self-esteem, and desire to quit medical practice/residency, as reported more by ECDs who worked longer hours in our study, are the result of burnout [6,26,27]. A positive correlation between burnout, and anxiety and depression has also been reported [28]. Burnout is a precursor to depression [12]. Our study corroborated these findings, as ECDs who had higher levels of burnout, also reported more anxiety and depression. Aside from anxiety and depression, physician burnout is associated with suicidal ideation, substance abuse, poor interpersonal and marital relationships, increased medical errors, and poor quality of patient care, amongst others [28]. Our study confirmed these findings, as male and lower cadre ECDs, who reported longer work hours, burnout, anxiety and depression, also alluded to having more suicidal ideation, alcohol consumption, tobacco smoking, and bad work life balance, compared to female and higher cadre ECDs.

The long work hours of Nigerian doctors, with the consequent high rates of burnout, anxiety, depression, and other adverse sequelae, may not be unconnected with massive medical brain drain currently bedevilling the country, which is the result of poor remuneration and working conditions, limited slots/opportunities for training, research, and career development, amongst others [6,28]. A consequence is the country’s current abysmal physician-to-patient ratio of 4:10,000 (a far cry from the WHO’s recommendation of 1:600), which has the potential of further worsening her already poor morbidity and mortality statistics [6,28].

A limitation of this study is the possibility of recall bias, as the data generated were based on self-reported information by the study participants. Another limitation is the inability to determine a true temporal link between cause and effect, as both were examined simultaneously, given the study design. These limitations notwithstanding, this study is the first in Nigeria to investigate key issues relating to the health, well-being, and burnout amongst Nigerian ECDs on a large scale–a nationally representative one. A probability sampling technique was adopted in this study, which gave all eligible participants an equal chance of participating in the study; hence, the possibility of selection bias was reduced to the barest minimum or absent. The study provides important data upon which relevant stakeholders can plan policies and interventions to address the health-related and psychosocial problems affecting ECDs in Nigeria.

## Conclusion

Our study clearly highlights the need to prioritize the health and well-being of Nigerian ECDs. It is beneficial for ECDs to adopt healthy lifestyles including regular exercises, cessation of smoking, and reduction of alcohol consumption. Hospitals should have facilities for physical exercises. There is an urgent need to address the systemic and individual challenges of Nigerian ECDs, with a view to providing solutions, and mitigating the negative impacts of these problems on the health and well-being of the ECDs, as well as on patient care and safety. Work hours of Nigerian doctors should be capped, and their working conditions, improved. Interventions that address burnout before anxiety and depression set in, would be beneficial.
